# Acute alcohol intoxication may cause delay in stroke treatment – case reports

**DOI:** 10.1186/s12883-019-1241-6

**Published:** 2019-01-29

**Authors:** Tamas Arokszallasi, Eszter Balogh, Laszlo Csiba, Istvan Fekete, Klara Fekete, Laszlo Olah

**Affiliations:** 1 Department of Neurology, Faculty of Medicine, University of Debrecen, Moricz Zs. krt 22, Debrecen, 4032 Hungary; 2 Cerebrovascular and Neurodegenerative Research Group of the Hungarian Academy of Sciences, Moricz Zs. krt 22, Debrecen, 4032 Hungary

**Keywords:** Alcohol intoxication, Stroke, Thrombolytic therapy, Diagnostic errors

## Abstract

**Background:**

The signs and symptoms of acute alcohol intoxication resemble those of vertebrobasilar stroke. Due to their shared symptoms including double vision, nystagmus, dysarthria, and ataxia, the differential diagnosis of alcohol intoxication and vertebrobasilar stroke may pose a challenge. Moreover, if alcohol intoxication and stroke occur simultaneously, the signs and symptoms of stroke may be attributed to the effects of alcohol, leading to delayed stroke diagnosis and failure to perform reperfusion therapy.

**Case presentations:**

Three cases of alcohol intoxication and stroke are presented. The first patient (female, 50 years old) had dysarthria, nystagmus and trunk ataxia on admission. Her blood alcohol level was 2.3**‰.** The symptoms improved after forced diuresis, but 5.5 h later progression was observed, and the patient developed diplopia and dysphagia in addition to her initial symptoms. Angiography showed occlusion of the basilar artery. Intraarterial thrombolysis was performed. The second patient (male, 62 years old) developed diplopia, dysarthria and trunk ataxia after consuming 4-units of alcohol, and his symptoms were attributed to alcohol intoxication. Two hours later, neurological examination revealed dysphagia and mild right-sided hemiparesis, which questioned the causal relationship between the symptoms and alcohol consumption. Cerebral CT was negative, and intravenous thrombolysis was administered. The third patient (male, 55 years old) consumed 10 units of alcohol before falling asleep. Three hours later, his relatives tried to wake him up. He was unresponsive, which was attributed to alcohol intoxication. When he woke up 8 h later, right-sided hemiparesis and aphasia were observed, and cerebral CT already revealed irreversible ischemic changes.

**Conclusions:**

Our cases show that alcohol consumption may interfere with stroke diagnosis by mimicking the signs and symptoms of vertebrobasilar stroke. Moreover, attributing the symptoms of stroke to alcohol intoxication may delay stroke diagnosis resulting in failure of reperfusion therapy. Based on our observations we conclude that stroke should be considered in the case of worsening symptoms, dysphagia, hemiparesis and disproportionately severe signs that cannot be attributed to the amount of alcohol consumed. In the case of ambiguity, ambulance should be called, and if stroke cannot be excluded, specific therapy should be administered.

## Background

Among patients presenting with acute neurological symptoms, it is crucial to decide in time whether they suffer from ischemic stroke and whether they are eligible for intravenous tissue plasminogen activator (IV tPA) treatment [1]. The short time window, however, may not always allow neurologists to establish a correct diagnosis, therefore it may be difficult to determine whether acute neurological signs are due to stroke or to other causes. Conditions with stroke-like symptoms but with other etiology than stroke are called stroke mimics [2–6]. One of the conditions that may mimic stroke is acute alcohol intoxication. Specifically, alcohol intoxication results in symptoms very similar to vertebrobasilar ischemia [7, 8], therefore the simultaneous occurrence of vertebrobasilar stroke and alcohol intoxication may result in the misdiagnosis of stroke. The common overlapping signs of vertebrobasilar stroke and alcohol intoxication including dizziness, dysarthria, nystagmus, ataxia with or without double vision and somnolence can easily be attributed to the acute effects of alcohol, and the misdiagnosis may lead to delayed stroke recognition and failure to administer appropriate therapy [9]. Moreover, as acute alcohol intake may be associated with inability to walk, hemispheric symptoms may also be overlooked by lay people with poor stroke awareness. Although there was one article which mentioned alcohol intoxication as a stroke mimic [5], to the best of our knowledge, no detailed description of this topic has been published in the scientific literature. During the past years, the acute effects of alcohol caused diagnostic challenges in some patients who presented with stroke symptoms at our department.

### Case presentations

**Fig. 1:**
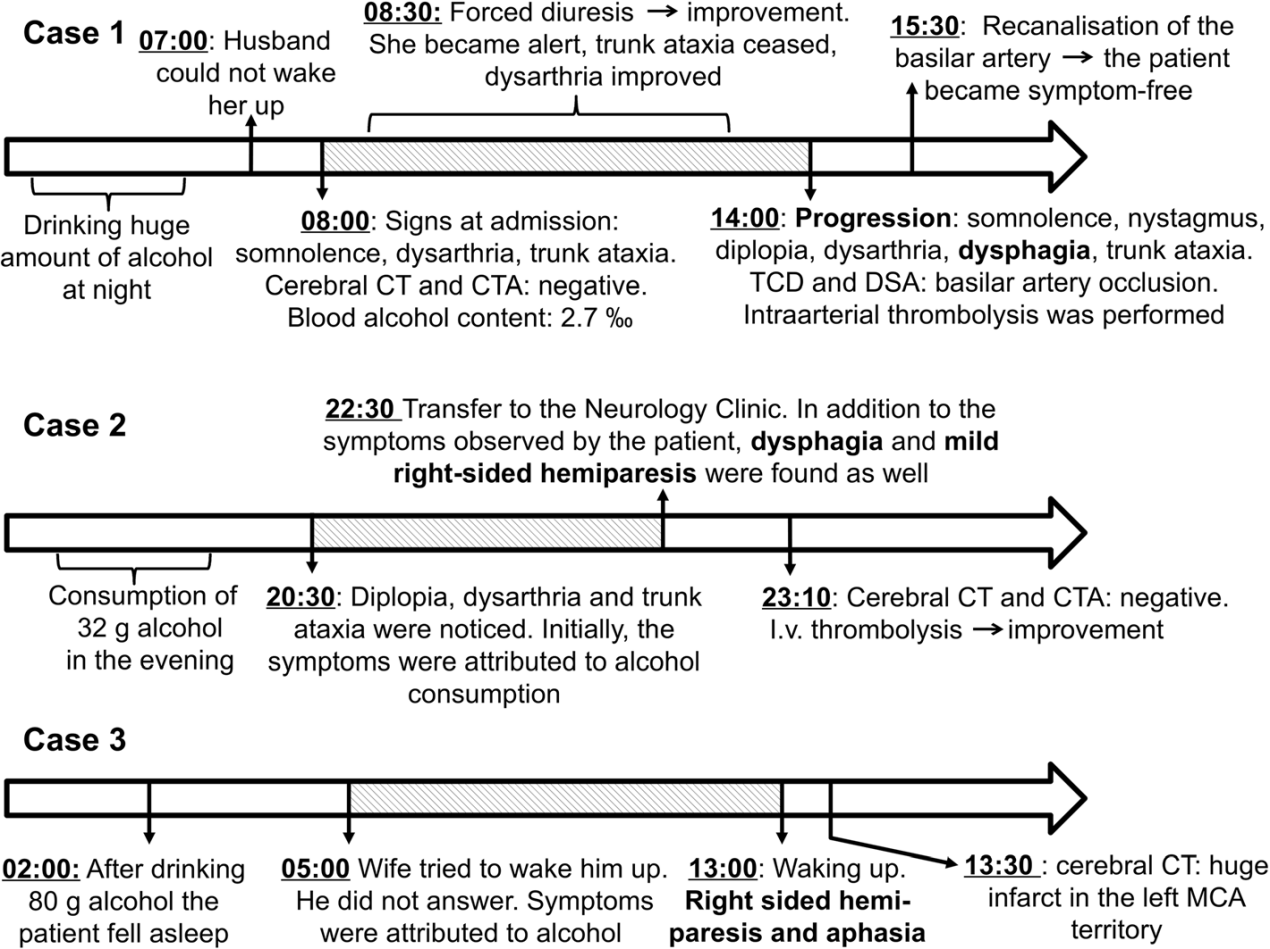
Timeline of the 3 cases. The delay of treatment due to alcohol intoxication is indicated by diagonal lines. Bold text highlights the symptoms which helped differentiate between stroke and alcohol intoxication. TCD: transcranial Doppler; DSA: digital subtraction angiography; CT: computer tomography; CTA: CT angiography; MCA: middle cerebral artery

**Fig. 2:**
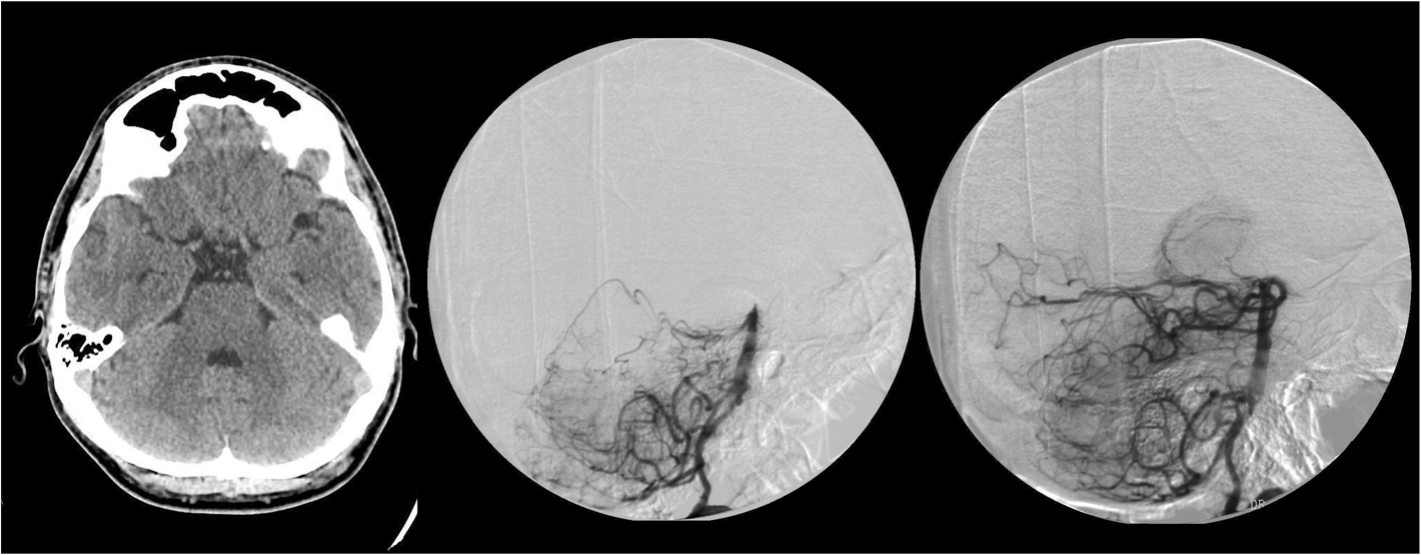
Case 1: Non-enhanced cranial CT showed no acute ischemic signs or hemorrhage. Digital subtraction angiography revealed basilar artery occlusion (A) and successful recanalisation after intra-arterial thrombolysis with 25 mg rtPA

Case 1 - A 50-year-old woman celebrated her birthday and consumed an unknown amount of alcohol. Her family was unable to wake her up the following morning (7 a.m.), therefore the patient was transferred to our department (Fig. 1). On admission, somnolence, moderate dysarthria, horizontal gaze-directed nystagmus, moderate trunk ataxia, and in-coordination were found. Her laboratory values showed moderate alcohol intoxication (Table 1). The symptoms were attributed to the effects of alcohol, therefore, after a negative CT and CT-angiography, forced diuresis was started (8:30 a.m.), and her clinical status was checked every hour. Initially, consciousness improved, the patient became alert, and dysarthria and ataxia ameliorated. However, early in the afternoon (2 p.m.), the control examination revealed worsening symptoms, she became somnolent again and developed severe horizontal nystagmus, double vision, dysarthria and dysphagia. Due to rapid progression, cerebral CT was repeated, which was negative again. Similarly, duplex ultrasound showed no stenosis of the carotid or vertebral arteries, however, transcranial Doppler (TCD) revealed high pulsatility index and low flow velocity in the basilar artery. Due to rapid progression and the sound suspicion of basilar artery occlusion, digital subtraction angiography (DSA) was performed. DSA showed basilar artery occlusion, therefore intraarterial thrombolysis was performed. After the administration of 25 mg rt-PA, the basilar artery was successfully recanalised (Fig. 2) and the symptoms rapidly improved. The control CT 24 h after the treatment showed no abnormalities. At discharge, the patient was symptom-free.

**Table 1:** Laboratory values of the patients

Laboratory values	Case 1	Case 2	Case 3
Glucose (mmol/L)	9,2	7,2	17,6
Creatinine (μmol/L)	78	65	58
CK (U/L)	47	78	133
AST (U/L)	15	18	14
ALT (U/L)	7	14	18
GGT (U/L)	10	21	38
CRP (mg/L)	UK	3,83	4,99
Ethanol (‰)	2,3	UK	< 0,1^a^
Ethanol (mg/dL)	230	UK	< 10^a^
White Blood Cell (G/L)	8,58	10,21	14,74
Hemoglobin (g/L)	135	150	161
Platelet (G/L)	294	277	281
INR	0,97	1	0,9

^a^ measured about 12 h after alcohol intake, *UK* unknown

Case 2 - A 62-year-old man consumed about 32 g ethanol (4 units) in the evening (Fig. 1). Before going to bed, his wife noticed his slurred speech, and the patient complained of double vision and trunk ataxia that was disproportionate to the amount of alcohol he had consumed. His wife attributed the symptoms to alcohol consumption; however, the patient disagreed. Therefore, paramedics were called who found mild right-sided hemiparesis and severe dysphagia in addition to double vision, dysarthria, and trunk ataxia. On admission to our department, the clinical examination confirmed these findings (NIHSS: 6 points). Cerebral CT showed no cerebral hemorrhage or infarction, therefore thrombolysis was performed within 3 h of the onset of symptoms. The control examination showed significant improvement, and the NIHSS evaluated 24 h after thrombolysis decreased to 1 point.

**Fig. 3:**
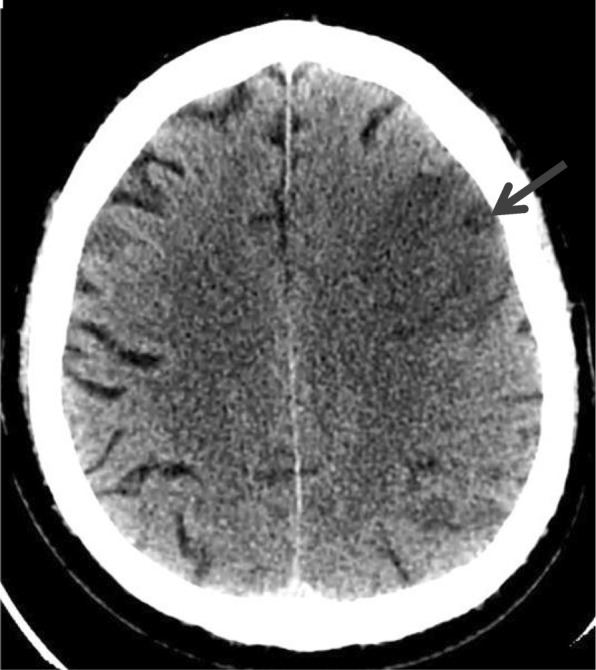
Case3: Non-enhanced cranial CT showed subacute ischemic lesion in the area of the left middle cerebral artery

Case 3 - A 55-year-old man consumed approximately 80 g etanol (10 units) during the night at a wedding ceremony and fell asleep at about 2 a.m. (Fig. 1). His relatives tried to wake him up early in the morning (5 a.m.), the patient opened his eyes, but could not speak. He seemed to be drunk, therefore the relatives attributed the signs to alcohol consumption and let him sleep back. Upon awakening in the early afternoon (1 p.m.), his relatives realized that he had facial asymmetry, mild right-sided weakness and speech disturbance. On admission, right-sided homonymous hemianopsia, paresis of the lower half of right side of the face, mild right-sided hemiparesis, and severe receptive and expressive aphasia were found. Urgent CT scan (1:30 p.m.) revealed a huge infarction in the left middle cerebral artery (MCA) territory (Fig. 3). Aspirin was administered and the risk factors were controlled. The neurological status did not change significantly.

## Discussion and conclusions

Based on worldwide data, the average alcohol consumption in 2010 amounted to 6.2 l of pure alcohol per person among people aged 15 years or older [10]. The highest alcohol consumption and the highest prevalence of heavy episodic drinking were shown in high-income countries.

In a systematic review and meta-analysis, acute alcohol ingestion was reported to cause an approximately two-fold increase in stroke as early as 1 h subsequent to alcohol consumption [11]. These results suggest that the simultaneous occurrence of alcohol intoxication and stroke is not a coincidence, because even moderate alcohol consumption is associated with an immediate increase in the risk of stroke. As the signs and symptoms of moderate-severe alcohol intoxication resemble those of vertebrobasilar stroke, stroke symptoms may be attributed to the effects of alcohol if the two conditions develop simultaneously. Moreover, heavy episodic drinking may also mask the signs of hemispheric stroke for people with poor knowledge of cerebrovascular diseases.

According to heteroanamnesis, all of our 3 patients undoubtedly drank significant amounts of alcohol before admission to our clinic. However, the time between alcohol consumption and blood alcohol level measurements varied (about 6 h in the first patient, and at least 12 h in the third patient), therefore the blood alcohol level measured after admission did not reflect the amount of ethanol consumed. Since the symptoms of alcohol intoxication and stroke overlapped and in the first and third patients the symptoms were only noticed after awakening, it was impossible to determine the precise onset of stroke. Based on our assessment, symptoms developed within 2 and 3 h of alcohol consumption in the second and third patients, respectively. The onset-to-door time was about 2 h in the second patient, but due to the delay in the recognition of stroke it was more than 8 h in the third patient. In the first patient, neurological symptoms were noticed within 5 h of alcohol consumption, however, these symptoms were most likely to be caused by alcohol intoxication, and obvious stroke symptoms developed later, already in the hospital.

Our observations show that acute alcohol intoxication may cause difficulties in differential diagnosis of stroke, especially if ischemia develops in the vertebrobasilar territory. Our cases suggest that thorough examination and observation are necessary to recognise stroke in the case of simultaneous occurrence of alcohol intoxication. Therefore, patients with alcohol intoxication require increased attention during emergency medical care. The first presented case underlines the importance of strict observation after alcohol consumption. Although cerebral CT and angio-CT were negative and the patient’s symptoms improved during forced diuresis, the cause of initial symptoms cannot be clearly determined. They could be due solely to alcohol intoxication or to alcohol intoxication with concomitant vertebrobasilar stroke with improving symptoms. Certainly, the worsening of clinical symptoms several hours after the initial improvement could not be explained by alcohol ingestion, but supported the diagnosis of stroke in this period of the condition. It remains unclear whether vertebrobasilar stroke was induced by alcohol and dehydration due to forced diuresis, or was independent of these conditions. This case underlines that patients with alcohol intoxication require increased medical attention, and emphasizes the need for repeated controls. Certainly, if MRI is available, diffusion-weighted imaging may help differentiate between alcohol intoxication and stroke [12, 13].

Our second case demonstrates that symptoms disproportionate to the amount of alcohol consumed and the asymmetry of symptoms are warning signs for stroke. It must be highlighted, however, that the patient’s relative would have attributed the severe stroke symptoms to the effect of small-moderate amount of alcohol. In the third case, the patient’s status was attributed to alcohol intoxication and therefore the stroke was not noticed in due time. The delay in stroke diagnosis resulted in late arrival at hospital beyond the thrombolysis time window, which eventually resulted in an unfavourable outcome. It is well-known that thrombolytic therapy helps dissolve blood clots and restore cerebral blood flow, and thus improves the outcome in acute ischemic stroke [14]. Based on our cases, it can be concluded that in the case of simultanoeus occurrence of stroke and alcohol intoxication, unusal alcohol-related clinical symptoms, such as dysphagia or hemiparesis, and disproportion between the symptoms and the amount of alcohol consumed may help the recognition of stroke in the background of symptoms. One of the lessons of this report is that in case of ambiguity, paramedics should be called and the patient should be transferred to a stroke unit. As patients with alcohol intoxication and stroke within the thrombolytic time window require increased medical attention, CT examination, angiography, and IV tPA treatment, these patients should be observed at a neurological ICU or in a well-equipped stroke unit. If the diagnosis of stroke cannot be excluded, diffusion-weighted MRI, if available, can be useful to determine the etiology of symptoms [13, 15]. However, it has to be emphasized that despite being the most sensitive technique for cerebral ischemia, even diffusion-weighted MRI may result in a false negative finding in stroke patients, especially in brain-stem strokes [13, 15]. If MRI is not available or negative, but clinical evaluation strongly suggests stroke, the worst should be assumed. In the case of dysphagia, asymmetry in symptoms including hemiparesis, or symptoms disproportionately severe to the amount of alcohol, ischemic stroke and rtPA treatment should be considered. In our patients, who arrived at the hospital in time and stroke diagnosis could be established within the therapeutic time window, thrombolysis resulted in a significant improvement of clinical symptoms. On the contrary, when stroke was not recognised, and therefore reperfusion therapy could not be initiated, no improvement was achieved. As thrombolysis in the case of stroke mimics is associated with no or only a minimal risk of hemorrhagic complications [16, 17], rtPA treatment can be suggested for patients with potential ischemic stroke and simultaneous alcohol intoxication, provided that the patient is eligible for rtPA therapy.

## Abbreviations

ALT: Alanine aminotransferase
AST: Aspartate aminotransferase
CK: Creatine kinase
CRP: C-reactive protein
CT: Computer tomography
DSA: Digital subtraction angiography
GGT: Gamma-glutamyl transpeptidase
INR: International Normalized Ratio
IV tPA: Intravenous tissue plasminogen activator (IV tPA)
MCA: Middle cerebral artery
MRI: Magnetic resonance imaging
NIHSS: National Institutes of Health Stroke Scale
rtPA: Recombinant tissue plasminogen activator
TCD: Transcranial Doppler
